# *ptxD/*Phi as alternative selectable marker system for genetic transformation for bio-safety concerns: a review

**DOI:** 10.7717/peerj.11809

**Published:** 2021-07-27

**Authors:** Richard Dormatey, Chao Sun, Kazim Ali, Sajid Fiaz, Derong Xu, Alejandro Calderón-Urrea, Zhenzhen Bi, Junlian Zhang, Jiangping Bai

**Affiliations:** 1 Gansu Provincial Key Laboratory of Aridland Crop Science/College of Agronomy, Gansu Agricultural University, Landzhou, China; 2 National Institute for Genomics and Advanced Biotechnology, National Agricultural Research Centre, Park Road, Islamabad Pakistan; 3 Department of Plant Breeding and Genetics, The University of Haripur, Haripur, Pakistan; 4 Department of Biology, College of Science and Mathematics, California State University, Fresno, CA, USA

**Keywords:** Genetic transformation, *ptxD*/Phi selection system, Selectable marker gene, Biosafety

## Abstract

Antibiotic and herbicide resistance genes are the most common marker genes for plant transformation to improve crop yield and food quality. However, there is public concern about the use of resistance marker genes in food crops due to the risk of potential gene flow from transgenic plants to compatible weedy relatives, leading to the possible development of “superweeds” and antibiotic resistance. Several selectable marker genes such as *aph, nptII, aaC3, aadA, pat, bar, epsp and gat*, which have been synthesized to generate transgenic plants by genetic transformation, have shown some limitations. These marker genes, which confer antibiotic or herbicide resistance and are introduced into crops along with economically valuable genes, have three main problems: selective agents have negative effects on plant cell proliferation and differentiation, uncertainty about the environmental effects of many selectable marker genes, and difficulty in performing recurrent transformations with the same selectable marker to pyramid desired genes. Recently, a simple, novel, and affordable method was presented for plant cells to convert non-metabolizable phosphite (Phi) to an important phosphate (Pi) for developing cells by gene expression encoding a phosphite oxidoreductase (PTXD) enzyme. The *ptxD* gene, in combination with a selection medium containing Phi as the sole phosphorus (P) source, can serve as an effective and efficient system for selecting transformed cells. The selection system adds nutrients to transgenic plants without potential risks to the environment. The *ptxD*/Phi system has been shown to be a promising transgenic selection system with several advantages in cost and safety compared to other antibiotic-based selection systems. In this review, we have summarized the development of selection markers for genetic transformation and the potential use of the *ptxD*/Phi scheme as an alternative selection marker system to minimize the future use of antibiotic and herbicide marker genes.

## Introduction

In genetic transformation, cells undergo a transformation treatment to introduce a foreign (exogenous) gene into the host genome. Transformation events are extremely low, so it is important to use efficient selectable marker genes along with the foreign gene of interest, which encode proteins that confer selection gain to transformed cells (Rosellini, 2012; Yang, Peng & Pan, 2019). A selectable marker gene has the properties for maximum selection of independent transformation events, minimum non-transformers or outliers, and easy detection of the marker gene and wide application in the plant species (Penna & Ganapathi, 2010). Usually, the selectable marker gene is positioned in the DNA vector construct along with the desired gene. In plant transformation, dominant selectable marker genes are developed based on genes that confer resistance to antibiotics or herbicides or have the ability to digest non-metabolizable substances (Miki & McHugh, 2004; Ravanfar et al., 2017). Resistance genes for antibiotics (hygromycin and kanamycin) and herbicides (bialaphos, glyphosate and the *bar*) have been used extensively as selection tools (Pandeya et al., 2018). However, many plant species are naturally resistant to antibiotics; for example, orchids are naturally resistant to antibiotics (Setiari et al., 2018). The sensitivity of various plant species to herbicides such as bialaphos and glyphosate cannot be overstated. Many commercially available genetically modified (GM) crops are bialaphos resistant (LibertyLink^®^), glyphosate resistant (Roundup Ready^®^), or have *bar* genes that confer resistance to the herbicide Basta (Breyer, Kopertekh & Reheul, 2014; Brown & Wernegreen, 2019). Herbicide-resistant genes show higher efficacy in performing plant transformation, but their use is limited by intellectual property restrictions and public perception (Nahampun et al., 2016). According to Ramessar et al. (2007) and (Molina-Márquez et al., 2019), there is a possibility of horizontal gene transfer associated with herbicide resistance traits in plant transformation processes, which draws the attention of researchers to search for an alternative selectable marker gene system. Public concern about herbicide-resistant markers, i.e., *bar* genes, is based on pollen flows from transgenic crops to compatible wild relatives, leading to the emergence of superweeds (Lal et al., 2020; Miki & McHugh, 2004; Pontiroli et al., 2007).

The use of antibiotic-resistant marker genes has been rejected by European Union (EU) and some other countries in both Asia and Africa because of the potential risk of horizontal gene transfer from plants to soil and gut microbes, which would lead to a large unintended spread of antibiotic-resistant genes (Pandeya et al., 2017). As a result, only a few thousand hectares (∼0.03% of world production) of genetically modified crops are grown in the EU (Brandt, 2003), likely reflecting European resistance to the technology. In contrast, food derived from GM crops is ubiquitous in the United States of America. In contrast, most animal feed used in Europe comes from imported plant material containing GM products. Similarly, GM cotton is used extensively as a raw material for the manufacture of clothing and related products (Key, Ma & Drake, 2008). Penna, Sági & Swennen (2002) pointed out that a major disadvantage of antibiotic resistant genes is that the selection scheme is based on the principle of negative selection, i.e., all non-transformed cells, which are much more numerous than the transformed cells, are successfully killed by the selection agent. Consequently, the majority of transformed cells cannot regenerate because dying or dead untransformed cells release growth inhibitors and lethal substances that interfere with the uptake of important elements from the growth medium by transformed cells. It has also been observed that some selection systems are more efficient than others for certain plant species and regeneration systems, simply because plants are sensitive to selection agents that are largely variable between plant species and tissues due to selection pressure (López-Arredondo & Herrera-Estrella, 2013; Wang et al., 2020). Therefore, an ideal marker system is needed that allows resistance to a lethal substance and, at the same time, can be converted into a substance essential for optimal growth and differentiation of cells in many plant species. The selection system allows to minimize or eliminate the risk of negative selection, as well as to recover false-positive clones that have escaped the selection system. In this scenario, it is important to design innovative and universally acceptable marker gene systems with higher transformation frequency. This can be achieved by stacking desirable genes to minimize both negative selection and the number of false-positive clones (Eckerstorfer et al., 2019; Pandeya et al., 2017).

The *ptxD* gene from *Pseudomonas stutzeri* WM88 encodes a NAD-dependent phosphite oxidoreductase (PTXD) (Costas, White & Metcalf, 2001). This enzyme is capable of catalyzing the oxidation of phosphite (Phi) to phosphate (Pi) and, in combination with Phi selection, offers the best option of a selection scheme for producing transgenic plants without the use of antibiotic- or herbicide-resistant genes, thus providing an innovative non-herbicidal mechanism for weed control (Changko et al., 2020; López-Arredondo & Herrera-Estrella, 2012; López-Arredondo & Herrera-Estrella, 2013). All plant cells require phosphorus (P) for reproduction, and cells harboring the *ptxD* are able to convert a nonmetabolizable P source (Phi) into a form of the compound (Pi) that they can readily incorporate into their metabolism. Selection of these cells should be easy over non-transformed cells that are unable to assimilate Phi as a P source. This unique property suggests the *ptxD*/Phi system as a possible general selection scheme to obtain efficient transgenic plants (González-Morales et al., 2020; López-Arredondo & Herrera-Estrella, 2013). In recent years, the *ptxD*/Phi system has been described as an effective selection marker in some important model organisms and crops such as *Arabidopsis* and tobacco (López-Arredondo & Herrera-Estrella, 2013), yeast (Kanda et al., 2014), sorghum (Che et al., 2018), cotton (Pandeya et al., 2017), rice (Manna et al., 2016), and *maize* Nahampun et al., 2016). It has been argued that this selection system is more beneficial than existing antibiotic and herbicide selection markers due to cost-effectiveness, efficiency and safety. This review summarizes the need for selectable marker genes for genetic transformation and the potential use of *ptxD*/Phi as an alternative selectable system to reduce the use of antibiotic and herbicide selectable marker genes in the future.

## Survey Methodology

We focused on developments in the link between biosafety concerns and genetically modified crops. We conducted a literature search using PubMed (https://www.ncbi.nlm.nih.gov/PubMed), Google scholar (scholar.google.cn), and Science web (http://www.webofknowledge.com). Keywords such as genetic engineering, gene transformation, *ptxD*/Phi selection system, selectable marker genes and non-selectable marker genes, food safety and biosafety concerns, effects of phosphite and phosphate on weeds and plant growth were used while related articles were extracted to categorize and summarize the potential effects of genetically modified crops on living organisms and their environment. Based on the checklist and database of PRISMA guidelines for systematic reviews (Liberati et al., 2009), among others, three hundred literature sources were screened as appropriate data sources after an initial data search, identification and removal of duplicates. Included in this review study were publications comprising 171 reviews, 85 research articles and 44 book titles and conference proceedings. The search was further restricted to references that included molecular breeding, molecular genetics, biotechnology, plant science, and agronomy. The main focus was on concerns about the biosafety of genetically modified (GM) crops, the use of novel molecular markers and alternative selectable markers for genetic transformation to produce GM crops. The GM crops may be better able to withstand biotic and abiotic stresses and also ensure food security and a safe environment.

### Need for marker gene selectable systems

Despite advances in gene transfer technology, transformation efficiency is very low, so that usually only a small proportion of transformed cells carry foreign DNA. Therefore, it is recommended to perform selection among the transformed cells or tissues from many non-transformed cells to regenerate genetically transformed plants (Penna & Ganapathi, 2010; Uddain & Subramaniam, 2020). Selectable marker genes are mainly genes used to recognize the transformed tissues or cells. These marker genes display a feature suitable for artificial selection of transformed tissues over non-transformed ones in a medium (Jekayinoluwa et al., 2020). Selectable marker genes are classified into many categories as they confer either positive or negative selection and selection is conditional or nonconditional depending on the presence of external substrates (Esland et al., 2018). According to Sundar & Sakthivel (2008), positive selection markers are used for genetic transformation to allow only transgenic cells to grow and develop and are necessary for efficient transformation, while negative selection marker genes lead to the death of transformed tissue. Depending on the functions of positive selection schemes, they can be categorized as conditional and non-conditional positive selection schemes (Esland et al., 2018). In conditional positive selection, a gene encodes a specific protein that confers resistance to a particular substrate that can be lethal to non-transformed plant cells or that only allows the development and differentiation of transformed tissues (García-Almodóvar et al., 2014). The conditional positive selection scheme includes antibiotics, herbicides, toxic and non-toxic chemicals, or a carbon source.

On the other hand, nonconditional positive selection schemes do not require external substrates but promote developmental selection and differentiation of transformed cells (Miki & McHugh, 2004; Rosellini, 2012). A typical example is the *ipt* gene, which promotes shoot growth by endogenously altering hormone levels in the plant. Babwah & Waddell (2000) and González-Morales et al. (2020) stated that once the selection technique does not depend on the substrate, it is called a nonconditional negative selection system. For example, the manifestation of a lethal protein such as a ribonuclease to remove certain cell types (Ma, 2005). When toxic gene activity requires a substrate to show toxicity, the method is called a conditional negative system (Babwah & Waddell, 2000; Wu et al., 2019a). Some examples of widely used positive selectable marker genes are *phosphomannose isomerase* (*manA*) and *xylose isomerase* (*xylA*) (Penna, Sági & Swennen, 2002; Zhang et al., 2019). Since these selectable marker genes regularly alter cell division and differentiation, there is a significant change in the development, morphology, and physiology of the transgenic plant (Table 1). Therefore, various tactics are necessary to reduce marker expression by using derivable promoters or generating plants without markers (Pandey et al., 2020; Penna, Sági & Swennen, 2002). The production of transgenic plants takes a long time, is labor intensive, expensive and obviously not an efficient process (Miki & McHugh, 2004; Tanner et al., 2020). This is the situation when dealing with important agricultural crops. The use of selectable marker genes speeds up the techniques of transformation and allows relatively early recovery of transgenic events (Lepais et al., 2020; Stoger et al., 2002). Selectable and visible marker reporter genes have little effect on the desired trait and also provide a valuable tool to determine the performance of transformed cells for the desired gene (Srivastava, 2019; Tuteja et al., 2012).

**Table 1:** Selectable markers for crop transformation. Some markers that have been used for GM crops over the years are summarized in this review. Details comprise the conferred phenotype, and examples of organisms for which the use of the marker has been described.

**Gene**	**Phenotype**	**Organism**	**Reference**
*merA*	Mercuric chloride	Peanut	Yang et al. (2003)
*pflp*	Erwinia chloride	Sweet pepper	You et al. (2003)
*atlD*	Arabital	Rice	LaFayette et al. (2005)
*OASAID*	5-methyltryptophan (5-MT)	Rice	LaFayette et al. (2005)
*xylA*	Xylose	Maize	Guo et al. (2007)
*ALS*	Bispyribac sodium	Wheat	Ogawa et al. (2008)
*dhlA*	1,2-dichloroethene	Rice	Moore & Srivastava (2008)
*ALS*	Bispyribac sodium	Soybean	Tougou et al. (2009)
*pmi*	Mannose	Sorghum	Gurel et al. (2009)
*pmi*	Mannose	Lettuce	Briza et al. (2010)
*aadA*	Streptomycin & Stectinomycin	Egg plant	Singh et al. (2011)
*dadA*	D-serine	Maize	Lai et al. (2011)
*tflA*	Toxoflavin	Rice	Koh et al. (2011)
*Bar*	Phosphinothricin	Soybean	Li et al. (2017)
*Epsps*	Hygromycin B phosphotransferase	Potato	Bakhsh et al. (2020)
*nptII*	Kanamycin	Potato	Bakhsh et al. (2020)

### Non-selectable marker genes in plants transformation

Non-selectable marker genes, also called reporter genes, encode molecules that are available visually or through biochemical controls and promote information about the cells or tissues that translate the inserted protein (Breyer, Kopertekh & Reheul, 2014; Olorunniji, Rosser & Stark, 2016). These genes are often used as “reporters” for gene expression by linking to other genes or promoters in GM products to express in the same manner as the linked gene or promoter (Lanigan, Kopera & Saunders, 2020; Miki & McHugh, 2004). They contain nontoxic proteins in plant materials and promote their physical assembly or controlled plant expression. The reporter genes *uidA* (*gus*) and *gfp* are commonly used to detect the activity of even a weak promoter (Cerezo et al., 2019). The *uidA* (*gusA*, or *gus*) gene is derived from *E. coli*, which converts the enzyme *β*-glucuronidase as a carbon and energy source (Elder et al., 2018; Porto et al., 2014). Expression of GUS from the inserted *uidA* gene can be detected in GM plant material using a GUS enzyme that produces a colored product when cleaved by GUS (Neumann, Kumar & Imani, 2020; Shrawat & Armstrong, 2018). The use of different growth media does not allow the sizing of the amount of protein available nor the visualization of the form of expression or distribution in the plant material (Banchi et al., 2019; Porto et al., 2014). The *uidA* gene with its associated protein is found in a wide variety of organisms, including *E. coli* and several other microbes, including other microorganisms of the digestive tract and soil bacteria (Porto et al., 2014; Riva et al., 2020). The work of GUS is common in all vertebrate tissues, with high activity in the kidney, liver and spleen (Xu et al., 2020). In turn, the activity of GUS is found in invertebrates such as mollusks, insects, and nematodes (Christiaens et al., 2018; Kumar et al., 2012). Moreover, low activity of GUS has been reported in more than forty different plant species and human food sources such as carrots, tomatoes and parsley etc (Mortensen et al., 2019). The *gfp* gene originates from the jellyfish (*Aequorea victoria*), which encodes green fluorescent protein (GFP) (Keutgen, Tomaszewska-Sowa & Keutgen, 2020) and emits a green light when exposed to blue or ultraviolet light. Zimmer (2002) revealed the main contribution of GFP helpers in bioluminescence of jellyfish. The green fluorescent protein is required as a gene expression marker for both GM animal and plant cells (Hoffman, 2015). Its expression in living tissue can be visualized by irradiation with ultraviolet or blue light without damaging the tissue. This allows observation of the intracellular location and mobility of related proteins in living cells (Franco-Bocanegra et al., 2019; Hanson & Köhler, 2001). Spontaneous modifications of the *GFP* gene sequence led to the emergence of a large number of variants with valuable properties (Su et al., 2019; Zimmer, 2002).

### Marker-free strategies or systems

Transformation without selectable marker genes (SMGs) is a suitable approach to obtain marker-free transgenic plants that increase consumer acceptance (Breyer, Kopertekh & Reheul, 2014). The development of GM plants without the use of SMGs has been reported in; *Arabidopsis*, barley, cassava, lime, potato, groundnut, tobacco, triticale (Chong-Pérez & Angenon, 2013; Manimaran et al., 2011; Schaart et al., 2011), alfalfa (Ferradini et al., 2011), apple (Malnoy et al., 2010), Prunus (Petri et al., 2011), orange (Ballester, Cervera & Pena, 2010), tomato (Xin & Guo, 2012) and wheat (Liu et al., 2011), (Table 2). Polymerase Chain Reaction (PCR) has been used in most cases to study the putative transformed events to detect the transgene (Breyer, Kopertekh & Reheul, 2014). Transformants selection can also be enhanced by the expression of a screenable marker: the *uidA* (GUS) or *GFP* gene, which makes the putative transformants a phenotypic asset associated with the gene expression of interest (GOI) (Bai et al., 2009), or it can be a direct screening of a product for the desired gene expression (Rukavtsova et al., 2013). Most studies on marker-free systems of experimental refinements have been used to improve some crucial factors, including the efficacy of DNA delivery technique and plant regeneration mechanism, for successful implementation of this process (Breyer, Kopertekh & Reheul, 2014). The refinements consist of improving treatment conditions to promote *Agrobacterium*-mediated transformation, e.g., vortex-mediated transformation of cold-treated seedlings (Rosellini & Veronesi, 2007; Weeks, Ye & Rommens, 2008), using *A. tumefaciens* strains that exhibit extremely high transformation efficiency (De Vetten et al. 2003). Most of these refinements take advantage of recent advances in identifying plant proteins and other factors within the transformation process and characterizing the molecular mechanism for successful T-DNA incorporation Anand, Vaghchhipawala & Mysore (2010); Barampuram & Zhang (2011).

**Table 2:** Examples of marker free crop plants detailing molecular techniques employed, marker genes used and crop species transformed are summarized in this review.

**Molecular techniques**	**Marker genes**	**Crop species**	**References**
Co-transformation	*Hpt and uidA*	Wheat	Permingeat et al. (2003)
Inducible site-specific recombination sys.	*Hpt, npt, codA, GUS*	Strawberry	Schaart et al. (2004)
Two-border binary vector	*Epsps-cp4, Epsp*	Maize	Huang et al. (2004)
Co-transformation	*Psy & phytoene*	Rice	Parkhi et al. (2005)
R/Rs recombination system	*CodA-npt II*	Potato	Kondrak et al. (2006)
Cre/lox site specific recombination	*GUS, npt II & gft*	Tobacco	Jia et al. (2006)
Cre/lox P	*Hpt, GUS & Gat*	Soybean	Li et al. (2007)
Marker-free binary vector	*Ipt*	Potato	Bukovinszki et al. (2007)
MAT system	*Ipt & npt II*	Cassava	Saelim et al. (2009)
Marker-free binary vectors	*Zmpsy or chitinase*	Peanut	Bhatnagar et al. (2010)
Co-bombardment	*Cry1B-Aa & hpt*	Rice	Kumar, Arul & Talwar (2010)
MAT system	*Ipt & wd*	Rice	Khan et al. (2011)
Co-transformation	*chi II, ap24, GUS & hpt*	Rice	Rao et al. (2011)
Marker-free binary vector	*At-CBF1 & npt II*	Tomato	Singh et al. (2011)
Cre/lox site-specific recombination	*Hph, gus & npt*	Rice	Akbudak & Srivastava (2011)
Cre/lox site-specific recombination	*Npt & GUS*	Rice	Khattri et al. (2011)
Ipt-type MAT vector	*GUS, ipt & chic*	Potato	Khan et al. (2011)
Marker-free & vector free cassette	*Acol*	Melon	Hao et al. (2011)
Ipt-type MAT vector	*Ipt and wd*	Tomato	Khan et al. (2011)
CRISPR-Cas9	*ZmTMS5*	Maize	Chen et al. (2018)
CRISPR-Cas9	*ARGOS8*	Maize	Shi et al. (2017)
MFTID	*Lox & bar*	Wheat	Cao et al. (2020)
Co-transformation	*Bar & GUS*	Wheat	Liu et al. (2020)
Marker steroid-inducible recombinase	*Npt II, gft & trfA*	Banana	Kleidon (2019)

In the development of genetically modified organisms (GMOs), many techniques have been elucidated to improve marker-free transgenic plants via the pollen tube pathway. This technique is widely practiced in China and effectively used in many crops such as maize, rice, wheat and cotton (Yang et al., 2009), melon (Hao et al., 2011) and soybean (Yang et al., 2011). The ovary-drip technique has also been used to achieve higher transformation in maize (Yang, Su & An, 2009). Marker-free production of GM products could be achieved via biolistic introduction of genes that bring unicellular microspores to pollen maturity and then use them for pollination (Aziz & Machray, 2003). Tuteja et al. (2012) reviewed several techniques or strategies that exclude marker-free product selection genes in transgenic development such as co-transformation, site-specific recombination, multi-auto transformation vector, system of transposition, and homologous recombination by (Puchta, 2000) and (Zubko, Scutt & Meyer, 2000). Among these techniques, co-transformation is the most widely used. Despite the continuous improvement of marker-free transgenes, the range of rescue of transformed events without the use of selectable marker genes remains highly susceptible to change and at least twofold or less than the use of antibiotic resistance marker genes (Breyer, Kopertekh & Reheul, 2014). In marker-free genetically transformed plants, selection events require a large number of GM transformants harboring the gene of interest (Bhatnagar et al., 2010). Another problem could be chimeric plants with only partially genetically transformed tissues due to lack of selection agents (Eckerstorfer et al., 2019; Li, Xie & Qiu, 2009; Joshi et al., 2009).

### Technique for the co-transformation and segregation of marker genes

**Figure 1:**
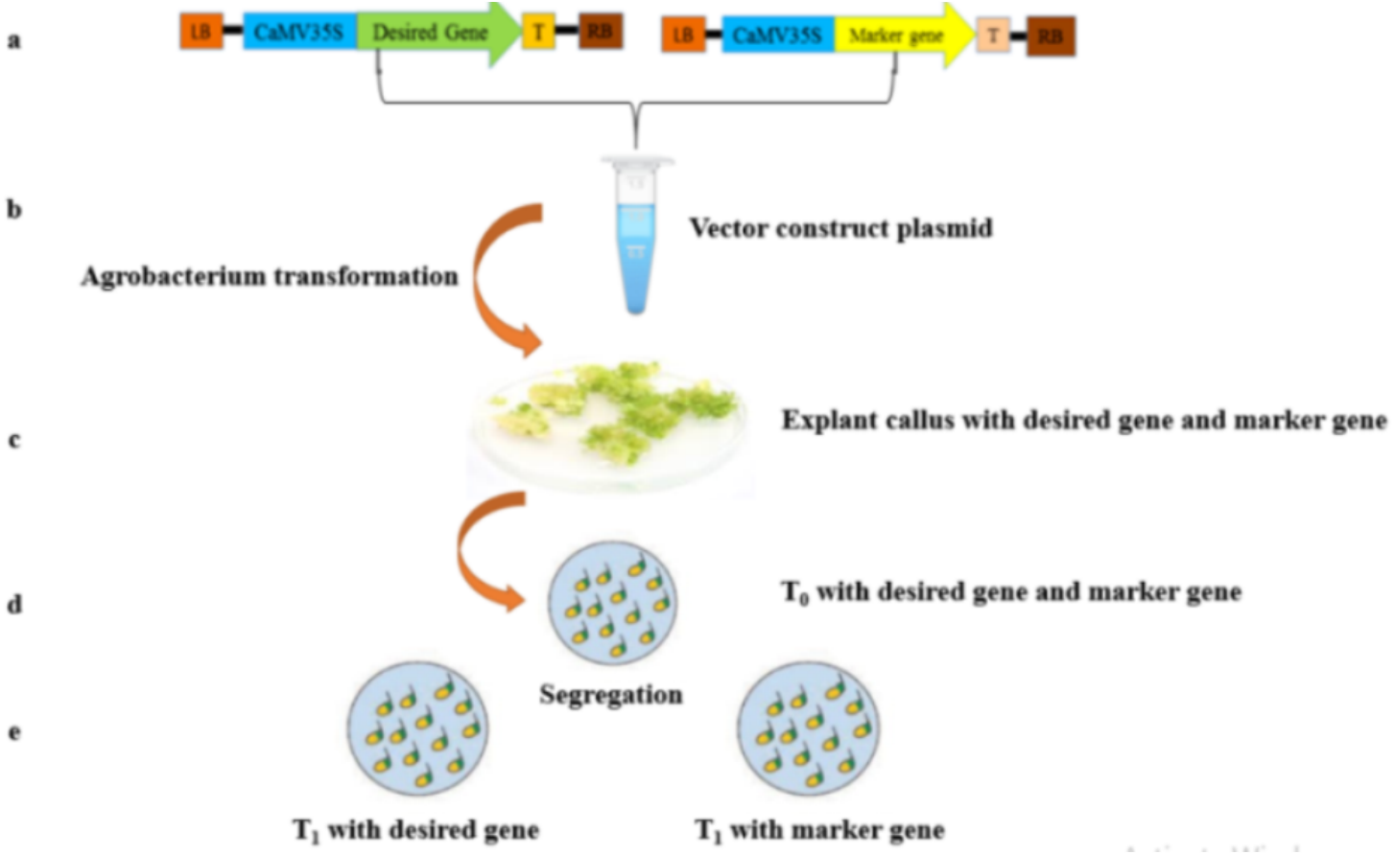
A scheme of genetic transformation procedure to produce marker free transgenic plants. (A) Two T-DNA sections physical diagram, showing desired gene and marker gene. (B) Putative transformed calli with the desired gene and marker gene. (C) Desired gene and marker gene harbored by T_0_ putative transgenic plant. (D) Segregating two different plants; T_1_: Potentially harboring the desired target gene and other T_2_ with only marker gene.

Co-transformation is a simple technique for obtaining transgenic products without marker genes (Breyer, Kopertekh & Reheul, 2014; Manimaran et al., 2011; Tuteja et al., 2012; Woo, Suh & Cho, 2011; Yau & Stewart, 2013). It involves the simultaneous transformation of a selectable marker gene and a desired gene from different T-DNAs into the genome prior to gene segregation in successive sexual generations. Four different approaches can be used in the co-transformation technique: (i) use of two different T-DNAs carrying two different plasmids in an *Agrobacterium* culture (Bahramnejad et al., 2019; McCormac et al., 2001; Sripriya, Raghupathy & Veluthambi, 2008); (ii) within an *Agrobacterium* culture, two different T-DNAs are placed on the same plasmid (Matthews et al., 2001; McCormac et al., 2001; Miller et al., 2002; Philips et al., 2019); (iii) the use of two T-DNAs in two different *Agrobacterium* groups (Quispe-Huamanquispe et al., 2019; Sripriya et al., 2011), and (iv) the two plasmids in one and identical biolistic delivery (Joyce & Sun, 2020; Kumar, Arul & Talwar, 2010; Prakash et al., 2009) (Fig. 1). The co-transformation technique involves the insertion of two T-DNAs into different genomic loci for segregation (Confalonieri & Sparvoli, 2019; Kausch et al., 2019; RamanaRao & Veluthambi, 2010). This technique offers unique advantages for the production of transgenic plants, i.e., it allows the simultaneous insertion of multiple genes of interest into a plant genome without many selectable marker genes, regardless of gene sequence (Miki & McHugh, 2004). In this technique, two to thirteen transgenes have been successfully incorporated simultaneously using biolistic gene gun (Low et al., 2018; Wu, 2007). The widespread use of biolistic methods may be critical to control multiple genetic traits using cloned genes, but extraction of marker genes from GM plants will be difficult (Ansari et al., 2020; Miki & McHugh, 2004).

Progress of GM plants without markers by co-transformation with *Agrobacterium* has been widely reported (Darbani et al., 2007; Manimaran et al., 2011; Narancio et al., 2020; Tuteja et al., 2012). This has been successfully used in crops like maize, rice, soybean and oilseeds. The method has also been used to develop first generation golden rice without hygromycin resistance marker gene (Al-Babili & Beyer, 2005). More recently, Holme et al. (2012) found that co-transformation has been used to generate cis-genic barley without markers. Theoretically, the technique appears to be simple and safe to generate non-marker GM plants compared to other techniques because it does not involve residual DNA sequences in GM products (Breyer, Kopertekh & Reheul, 2014). As an advantage of co-transformation by *Agrobacterium* compared to biolistic transformation, it is reported that the genes co-transformed by *Agrobacterium* are usually incorporated into the plant genome at different loci. Thus, different marker genes can be easily separated from the desired genes, which facilitates the generation of transgenic plants without markers (Ebinuma et al., 2001). Essentially, the co-transformation associated with the removal of selectable marker genes only applies to sexually reproducing species, but not to asexually propagated plants such as potato, date palm, sugarcane, and some woody plant species (Darbani et al., 2007; Manimaran et al., 2011; Parray, Mir & Shameem, 2019; Tuteja et al., 2012). Breyer and colleagues emphasized that the use of non-selectable marker genes seems to be the easier way to produce GM crop with reduced presence of introduced DNA sequence and associated biosafety issues (Breyer, Kopertekh & Reheul, 2014). This protocol has been successfully applied to the production of *Bt* cotton using the pollen tube pathway technique or the genetically transformed potato AV43-6-G7 through agrobacteria-mediated transformation. However, the main disadvantages of non-marker transformation techniques are the poor recovery rate of transformants, high labor, time and cost requirements, and the use of a large number of regenerated plants for PCR analysis (Breyer, Kopertekh & Reheul, 2014; Confalonieri & Sparvoli, 2019). In addition, this technique requires high transformation and regeneration efficiencies that are typically genotype-based, limiting its application to very limited organisms. The technique is not feasible on a commercial scale for generating crops with desired genes and agronomic traits, except that the efficiency of transformation protocols is significantly increased (Jansing et al., 2019; Tuteja et al., 2012).

### Bio-safety concerns related to GM crops

The influence of GM plants on production and cropping patterns of agricultural species worldwide is becoming increasingly popular in modern times (Kadoić Balaško et al., 2020). However, the widespread adoption of GM plant varieties and their cultivation have raised notable concerns about biosafety issues in some parts of the world (Luna & Dowd-Uribe, 2020; Stewart, 2001). The most common biosafety concerns relate to the direct or indirect flow of toxic origin transgenes to non-target species; the possibility of unforeseen effects in transgene-environment interactions and leakage of transgenes from GM plant varieties into their weedy relatives is the most intense debate worldwide (Beacham, Sweet & Allen, 2017; Lu & Snow, 2005; O’Callaghan et al., 2005; Oliveira et al., 2007; Souza et al., 2019). In line with these concerns about GM products, the Food and Agriculture Organization (FAO), the World Health Organization (WHO), and the Organization for Economic Cooperation and Development (OECD) and other nations, through expert consultations, have accepted the following relevant health requirements that should be considered when reviewing a novel food (Krebs, 2000). These include: (i) the modified host organism should be described, including information on the composition of nutrients (antibiotics, toxins and allergenic potential, and other important changes during usual processing.); (ii) the organism used as a donor should be well described, with information on possible toxicity and related allergens, and the genes used and their products should be free of any public health risk; (iii) molecular description of the genetic transformation, with description of the modification process; (iv) documentation of the primary and any secondary gene products described, with an explanation of the foreign gene characteristics; (v) evaluation of the safety of the anticipated novel substances in food, with an assessment of any toxins formed directly by the modification; (vi) evaluation of novel food allergy possibilities; and (vii) evaluation of unintended effects on dietary composition and assessment of changes in nutrient enrichment. Considering the regulatory concerns of various countries around the globe towards GMOs, it is imperative to develop new and widely accepted marker gene systems to introduce more desirable genes for the production of the future GM crops. Therefore, the selectable markers i.e., *ptxD*/Phi are proposed as an alternative scheme for genetic transformation to develop safer crops.

### The *ptxD*/Phi; alternative selectable marker for crops genetic transformation

To minimize the use of antibiotic or herbicide resistance genes, several alternative transformation systems have been proposed by bioscientists (Hu et al., 2016; Miki & McHugh, 2004; Patterson et al., 2019). Some of these markers are easily identified by the human eye and have been used as alternatives to antibiotic or herbicide markers (Boscaiu et al., 2019; Naing et al., 2015). For example, the anthocyanin gene is used as a substitute for strawberry, apple, and potato transformation selection of kanamycin (Anwar et al., 2018; Kortstee et al., 2011), mainly because anthocyanin is known to be an anti-cancer agent that may also provide health benefits (Breyer, Kopertekh & Reheul, 2014; Diaconeasa et al., 2020). The other most commonly used positive marker genes are the *xylA* gene from *Streptomyces rubiginosus* (Barampuram & Zhang, 2011; Tran et al., 2020) and *Thermo-anaerobacterium thermosulfurogenes* (Shrawat & Lörz, 2006), *Xylose isomerase* catalyzes the isomerization of xylose to d-xylulose, which can be used as a carbohydrate base in plant cells. These genes have been successfully used to develop transgenic potato, tobacco and tomato. However, d-xylose produces carbon, which is toxic to plant cells, so selection requires culture on a precise mixture of sucrose as well as d-xylose (Ghazanfar & Irfan, 2020; Haldrup, Noerremark & Okkels, 2001). Another positive marker gene, *Phosphomannose isomerase* (PMI), is considered credible and has been extensively studied (Penna, Sági & Swennen, 2002; Singh et al., 2019). Plant cells contain endogenous hexokinase that can convert mannose to mannose-6-phosphate. Therefore, cells expressing PMI can convert mannose-6-phosphate to fructose-6-phosphate, which can be used as a carbohydrate source in the plant (Penna, Sági & Swennen, 2002; Singh et al., 2019). Selection on the *PMI*/mannose scheme has been reported in the transformation of sugarcane, sorghum, sugar beet, rice, and maize (Penna, Sági & Swennen, 2002; Shi et al., 2020; Wu et al., 2019a). According to Lynch et al. (2019), this selection system is based on alternative carbon origin using mannose as a selection agent, where a specific mannose mixture is cultivated with one of the other sugars, which must be adapted to both the selection stage and the plant species to achieve optimal transformation efficiency.

Recently, the phosphite oxidoreductase (*ptxD*) gene, a unique dominant marker for selection in transformation of plants and other organisms, has been reported (Heuer et al., 2017b; Hirota, Motomura & Kuroda, 2019; López-Arredondo & Herrera-Estrella, 2013). For example, the rice *Actin2* promoter (*OsAct2P*) and terminator (*OsAct2T*) were used to clone the *ptxD* expression cassette, which was then incorporated into the pMDC99 vector for rice transformation (Manna et al., 2016). The constitutive promoter (rice actin2) was selected for a higher level of transgene expression, allowing rapid oxidation of Phi after it is absorbed by Pi transporters in the plant. Overall development, chlorophyll assembly, root development, PS-II activity, and Phi and Pi assembly were comparable in Phi-metabolizing transgenic plants developed in Phi media as the major P fertilizer compared with plants fed Pi. Phi-mediated growth inhibition has been observed in a variety of plant species in previous studies (Carswell, Grant & Plaxton, 1997; Hirosse et al., 2012; Thao et al., 2008a; Ticconi, Delatorre & Abel, 2001). For instance, application of Phi severely restricted root development of onion (*Allium cepa*) (Sukarno, Smith & Scott, 1993) and *Brassica nigra* (Carswell, Grant & Plaxton, 1997). In spinach, a decrease in the phosphate:phosphite ratio resulted in a decrease in shoot dry weight (Thao et al., 2008a). Similar results were observed in *Brassica napus* var Peruviridis (Thao et al., 2008b). Development, length and dry weight of sweetpotato plants were reduced with increasing Phi treatment (Hirosse et al., 2012). In addition, rice root and shoot biomass, root hair formation, and chlorophyll formation decreased when Phi concentrations in the media were increased (Manna et al., 2015). Ticconi, Delatorre & Abel (2001) observed similar results in *Arabidopsis*. However, López-Arredondo & Herrera-Estrella (2012) observed that Phi metabolizing transgenic *Arabidopsis* and tobacco plants expressing the bacterial *ptxD* gene resulted in improved phenotype and physiology in terms of biomass accumulation, yield as well as Phi build-up. This finding supported the hypothesis of Manna et al. (2016) that when rice plants evolved the ability to utilize Phi, there was a significant increase in growth, phenotype and physiology of rice seedlings, with better Pi build-up and lower Phi accumulation compared to wild rice plants grown in related Phi concentrations.

Furthermore, Che et al. (2018) evaluated PTXD/Phi in a ternary vector with pPHP70444 containing the *ptxD* gene driven by a maize ubiquitin promoter and intron. PTXD/Phi selection was imposed, resulting in more stringent selection and healthy callus growth. This resulted in transformation performance of up to 6% with no detectable outliers and a level of quality events of 47%. A novel non-antibiotic and non-herbicidal sorghum transformation scheme was developed using PTXD as a selectable gene. Despite the lower transformation frequency in sorghum, the PTXD/Phi selection scheme is a promising transformation system that can further increase the efficiency of selection in crops through codon optimization of the *ptxD* gene in monocotyledons (Che et al., 2018).

A similar study on cotton transformation using the same selection method showed that the *ptxD*/phosphite selection system provides a high-quality, effective, and simple method to produce GM cotton plants and addresses several concerns regarding the use of antibiotic- and herbicide-resistant genes in the development of transgenic organisms (Pandeya et al., 2017). The *ptxD* gene can be used to select transformed cells and produce transgenic cotton plants using a selection medium with Phi as the main source of P (Pandeya et al., 2017). A total of 3.43% transgenic events were obtained with the *ptxD*/Phi selection scheme, compared to only 0.41% with *bar*/phosphinothricin (PPT) selection. The *nptII*/kanamycin and *hpt*/hygromycin systems had event recovery rates of 2.88 and 2.47%, respectively (Pandeya et al., 2017). These results indicate that “clean” *ptxD*-expressing callus genotypes can be obtained by replicating subcultures and selecting the most evolving cultures without the use of visible, screenable marker genes. The selection mechanism under *ptxD*/Phi compared to selection based on the use of antibiotic and herbicide resistance genes in cotton transformation showed that *ptxD*/Phi selection produced a higher percentage (97%) of transformants compared to *nptII*/kanamycin and *hpt*/hygromycin using PCR analysis of putative transformants of regenerated events harboring the *ptxD* gene (Pandeya et al., 2017). It was observed that while Phi selection does not completely kill cultures lacking either the *ptxD* transgene or its expression, it does increase the growth of cells expressing the gene for selection (Nahampun et al., 2016; Pandeya et al., 2017). These results are in agreement with those of (López-Arredondo & Herrera-Estrella, 2012), who conducted a study to investigate the ability of medium containing Phi (1 mM) in the selection of *ptxD*-expressing organogenic shoots regenerating from leaf disks of tobacco after *Agrobacterium-* mediated transformation. Nahampun et al. (2016) transformed two maize genotypes: type I and Type II callus cultures separately with the same bacterial *ptxD* gene, and the transformants were subsequently selected on medium containing KH_2_PO_3_.

The novelty of *ptxD*/Phi selection is that the system allows the conversion of a toxic compound (Phi) into a valuable compound (Pi) for optimal plant growth. In this selection system, escape events are minimal and the nutritional value for the plant is also provided (López-Arredondo & Herrera-Estrella, 2012; López-Arredondo & Herrera-Estrella, 2013; Pandeya et al., 2018). The *ptxD*/Phi scaffold offers several advantages over currently used systems: (i) the Phi salts contain sodium and potassium, which are affordable and easily available from different companies, (ii) the phosphite salt is harmless to humans and animals, therefore no special precautions are required for its use, (iii) the phosphite salts are well soluble in thermal water and photostable, which makes them constant selection agents in tissue culture and greenhouses, and (iv) the *ptxD*/Phi method is suitable as a selection system in the greenhouse using inert substrates or different low-Pi soils by adding Phi in the soil or fertilizing to promote high-density experiments (Achary et al., 2017; López-Arredondo & Herrera-Estrella, 2012; López-Arredondo & Herrera-Estrella, 2013). Comparing the *ptxD*/Phi selection scheme to other selection systems, only the Pi ion in the culture medium needs to be replaced with a Phi ion to efficiently select cells expressing the *ptxD* gene, thereby eliminating all steps to modify the gene. In this scenario, the *ptxD*/Phi framework is considered to be a precise and beneficial addition to the plant transformation toolbox, eliminating the dependence on antibiotic and herbicide resistance genes, making it easier to overcome some problems related to the introduction of GM plants. Moreover, *ptxD*/Phi technology symbolizes one of the most exciting transforming selection systems of today, which not only provides an efficient way to generate transgenic plants, but also helps to address a number of concerns related to the use of antibiotic and herbicide resistance genes, as well as biosafety concerns in transgenic development.

However, plants treated with phosphite (Phi) rapidly accumulate Phi within their cells (McDonald, Grant & Plaxton, 2001). Phosphite is phloem mobile and accumulates in sink tissues (Leong, Lu & Chiou, 2018). Since Phi is not metabolized by plants, it remains in tissues for a long time and consequently disrupts the signal transduction chain that allows plants to detect and respond to Pi deficiency at the molecular level, thereby amplifying the negative effects of Phi (Mehta et al., 2021; Trejo-Téllez et al., 2019). In Pi-deficient tomato plants cultured in the presence of Phi, the expression of Phi-inducible genes such as *PT1* and *PT2* (high-affinity Pi transporters), *PS2*, and *TPSI1* (novel genes) was strongly repressed. Moreover, Phi accumulates massively in the cytosol and blocks Pi efflux from the vacuole, while subsequent uptake of Pi into cells triggers an enormous transfer (Gómez-Merino & Trejo-Téllez, 2016). Phi is transported from the cytosol to the vacuole. Pi deficiency symptoms can be exacerbated by this suppression of Pi efflux from the vacuole, leading to increased programmed cell death in Pi-deficient or *ptxD* non-transgenic plants (Gómez-Merino & Trejo-Téllez, 2015).

### The *ptxD*/Phi technology; a novel strategy for weeds control

To meet the food needs of the ever-growing population, the focus is on increasing crop productivity under challenging conditions (Catalá & Salinas, 2018). The main problem for farmers in this situation is to ensure optimal nutrient levels in the soil and control weeds (Catalá & Salinas, 2018). Typical weed control measures are tedious practices such as hand weeding, hoeing or ecologically aggressive methods such as tillage. The earlier discovery and application of herbicides such as 2,4-dichlorophenoxyacetic acid and Roundup eradicated most weeds growing in the soil (Armengot et al., 2015; Troyer, 2001). The overuse of these herbicides has led to the emergence of numerous herbicide-resistant weeds in fields. There are previous reports of 254 herbicide-resistant weed species, of which 41 are resistant to glyphosate Heap (2018). This worrisome condition has called for persistent research to find new strategies to control weeds in cereal fields. According to Pandeya et al. (2018), selective fertilization of transgenic plants carrying *ptxD* gene with Phi is an effective means to control the growth of weeds. The *ptxD* transgenic plants are able to metabolize Phi by converting it to Pi, a mechanism that enables the transgenic plants to outcompete many dicotyledonous and monocotyledonous weed species in both natural soils and substrates. More importantly, *ptxD*/Phi technology suppressed the growth of *Amaranthus palmeri*, a glyphosate-resistant weed that has devastating effects on many crops (Smith, Baker & Steele, 2000). In a study conducted on artificial inert soils and natural soils, Phi fertilization processes severely hindered *A. palmeri* emergence (Pandeya et al., 2018).

**Figure 2:**
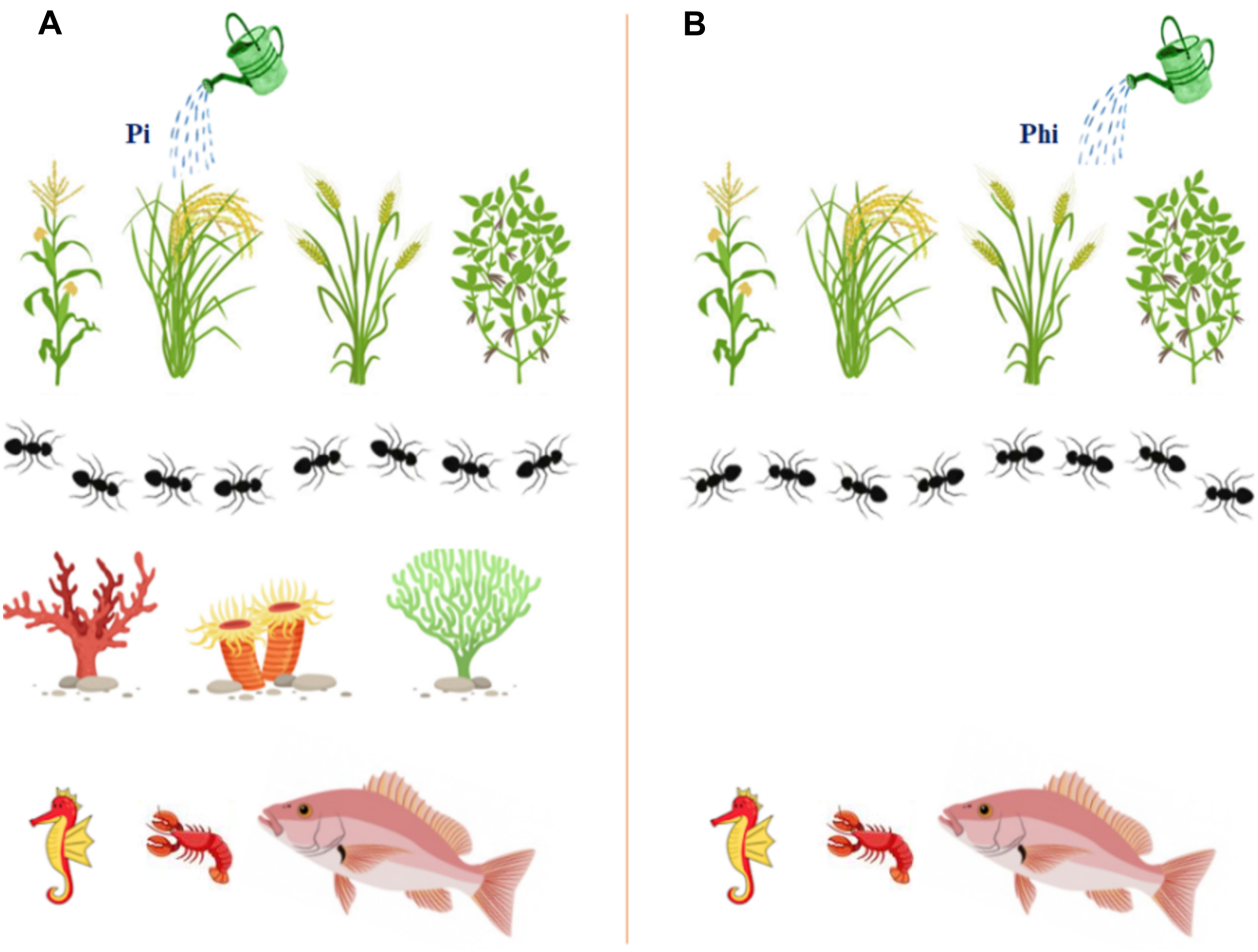
The graphical illustration to display the safety of *ptxD*/Phi selection system on different organisms and the environment, and showing the added advantages to enhance transgenic plants growth. (A) Phosphate (Pi) fertilizer (B) Phosphite (Phi).

The *ptxD*/Phi system for weed control has been previously validated for other plant species, such as tobacco, which harbors the gene and effectively suppresses many weeds such as *B. distachyon*, *I. purpurea*, *Brachiaria plantaginea*, and *Amaranthus hybridus* when Phi was used instead of Pi (López-Arredondo & Herrera-Estrella, 2012). The *ptxD*/Phi system proved to be an efficient weed control tool in trials conducted under field conditions in Argentina (Heuer et al., 2017a). In another recent study on rice, Phi showed a positive effect against *Amaranthus spinosus* as a selective pre-emergence weed control (Manna et al., 2016). In addition, foliar application of Phi was more efficient in controlling widespread weeds such as *Phyllanthus niruri*, *Euphorbia hirta*, *Portulaca oleracea* and *Chloris barbata*, a monocot plant. The *ptxD*/Phi mechanism for weed control was not only effective but also provided an innovative molecular approach to reduce the overdependence on phosphate as a P source for current agriculture by expressing the *ptxD* gene in tobacco and *Arabidopsis* (López-Arredondo & Herrera-Estrella, 2012). The data confirmed that *ptxD* transgenic plants required 30–50% less phosphorus in greenhouse situations when supplemented in the form of Phi instead of Pi to achieve a biomass equivalent to the wild form fertilized by Pi (López-Arredondo & Herrera-Estrella, 2012). Other advantages include the lower fixation of Phi in the soil compared to Pi and the fact that few microbes can use Phi as a source of P (Heuer et al., 2017a; Lopez-Arredondo et al., 2014; McDonald, Grant & Plaxton, 2001). In addition, soil microbes, including algae, cannot utilize Phi as a major source of P. When phosphite is released from fields into water bodies, algal species cannot utilize it as a source of P, minimizing the effect of P fertilization to promote toxic algal blooms (Loera-Quezada et al., 2016) (Fig. 2). The effects of water eutrophication and toxic algal blooms have resulted in more than 400 lethal zones in many parts of the world’s oceans, covering more than 250,000 square kilometers of ocean surface (Hardy et al., 2016; Schmale et al., 2019). For this reason, the *ptxD*/Phi scheme can offer important ecological gains by reducing eutrophication of water bodies as well as hypoxia due to Pi and N fertilizer runoff into river bodies. Finally, it should be noted that Phi poses no risk to human or animal health and is massively used as an efficient fungicide in crop production (Achary et al., 2017). Thus, phosphite has a direct effect on phytopathogenic fungi by inhibiting mycelial proliferation and reducing conidiogenesis of *Fusarium* sp. isolated from the rhizosphere of plants (Solis-Palacios et al., 2021). In addition, Phi can act indirectly by activating the innate defense mechanisms of plants to limit pathogen growth (Cerqueira et al., 2017). Phi can also activate host defense genes, which helps plants ward off disease (Rampersad, 2020) and directly suppress the growth of pathogens such as *Phytophthora* (Huang et al., 2018). Phi is expected to interfere with the metabolic mechanisms of *Phytophthora* associated with phosphorus uptake. Phosphite accumulates around root tips in the area colonized by mycorrhizal fungi, which is transmitted through the phloem (Hunter, 2018). When phosphite accumulates in the system, it can induce necrosis causing damage to fine roots, resulting in the loss of ectomycorrhizal and arbuscular mycorrhizal production sites in plant species that form symbiotic interactions with mycorrhizal fungi (Plaxton & Carswell, 2018). Application of this Phi may damage the mycorrhizal interface because mycorrhizal fungi extend the lifespan of fine roots and active fine roots serve as a metabolic sink for photosynthates (and thus phosphite). Furthermore, any damage to immature roots would alter the pattern of root exudates and may lead to changes in the soil microbiota (Jorgensen, 2014), which may indirectly affect mycorrhizae (Jorgensen, 2014). Wu et al. (2019b) hypothesized that Phi treatment significantly increases isoflavonoid production, which explains why Phi-treated plants are more tolerant to various biotic stressors. Nevertheless, the expected expansive implementation of the *ptxD*/Phi program in the near future would require further studies to assess the likely and potential environmental impacts of Phi operation.

The transgenic plants carrying the *ptxD* gene can use phosphite (Phi) as the sole source of phosphorus (P) by converting Phi to phosphate (Pi) to achieve optimal growth and development, which can lead to higher yields (Fig. 2). Plants, soil and aquatic microbes cannot metabolize phosphite, so weeds cannot compete with crops for available nutrients, nor can aquatic life. Phosphite moves in water bodies and has no significant growth effect on aquatic microorganisms. Studies have shown that aquatic microorganisms such as algae, *Ulva lactuca*, *Chlamydomonas reinhardtii*, *Botryococcus braunii*, and *Ettlia oleoabundans* are unable to use Phi as a source of P and therefore have no negative effects on aquatic life (Lee et al., 2005; Loera-Quezada et al., 2015). However, accumulation of significant amounts of Pi in water bodies triggers algal blooms. This compound is leached from croplands that are heavily loaded with phosphate fertilizers. Rain is a means of washing these leachable compounds from the soil surface into water bodies, which eventually end up in large reservoirs such as lakes and oceans. Drainage systems discharge these nutrient components into rivers, and untreated raw sewage flows into water bodies and induces algal blooms.

Much attention needs to be paid to measuring phosphate levels and monitoring water quality, as well as regulating algal phytoplankton dynamics against long-term environmental impacts. Phosphate enrichment of algae has been widely studied to determine their beneficial uses (De-Bashan & Bashan, 2004). The consequences of algal blooms (Mallin & Cahoon, 2020) include threats to human health/life: algal blooms contain toxins that reduce the suitability of water for human consumption. This claim is based on the fact that algal blooms in water contribute to rapid contamination of water, which poses a threat to human health and leads to severe irritation, itching and skin diseases when such contaminated water comes into contact with human skin. Fish and other aquatic life depend on dissolved oxygen in water. However, in the case of plants, heavy reproduction and dense growth in a very short period of time leads to increased competition for oxygen, resulting in an imbalance in the aquatic environment and suffocation of aquatic animals such as fish. Fertilizer contamination can lead to dead zones with little or no oxygen in the water, a scenario that makes it difficult for aquatic life to survive. This condition is called hypoxia. Oxygen-depleting algae blooms cause these dead zones as they die and decompose, which can lead to a large death of aquatic life, making the algae bloom region a dead zone with dead animals and plants. The resulting foul odor can affect the remaining aquatic life and drive them away further. Phosphate fertilization allows weeds to compete with crops such as cotton, cowpea, and other crops for the available phosphorus source, limiting their growth and development. Much of Pi is metabolized by microbes in the soil, making it unavailable to plants. Importantly, the soil microbiota and aquatic microbes cannot metabolize Phi, which greatly increases its availability to plants, which in turn reduces the risk of eutrophication of water bodies.

### *ptxD* /Phi as selectable marker system in microorganisms for biological contamination control in cultivation systems

Mineral nutrient availability is a major limiting factor for microalgal culture production and conservation (Juneja, Ceballos & Murthy, 2013). Researchers and private companies have considered transgenic microalgae and cyanobacteria as highly potent green cell factories for the production of valuable bioproducts (Ng, Keskin & Tan, 2020). Therefore, the promoter (Pccg6) has been used to stimulate the expression of the bacterial phosphite oxidoreductase PTXD, which allows *Trichoderma atroviride* phosphite (Phi) as a major phosphorus source (Carreras-Villase nor et al., 2020). Recent research has shown that PTXD expression in both plants and microorganisms enables a very challenging climate that promotes the production of genetically modified organisms while limiting the production of diverse, weedy combinations of organisms (plants, fungi, microalgae, and bacteria) that are unable to metabolize Phi (Heuer et al., 2017a; Loera-Quezada et al., 2016; López-Arredondo & Herrera-Estrella, 2012; Pandeya et al., 2018). *Trichoderma ptxD* transgenes containing constructs of the *ccg6OPT* or *pki1OPT* genes showed positive phenotype developmental traits. Thus, the transgenic strain exhibits comparable growth kinetics on Pi and Phi containing media, suggesting that PTXD expressions in *Trichoderma* do not have destructive effects on its physiology (Carreras-Villase nor et al., 2020).

The use of PTXD/Phi has been shown to be an effective technique to control contaminating species (e.g., *Kluyveromyces marxianus* CBS 6556, *S. cerevisiae* Ethanol Red) in the fermentation of a number of *S. cerevisiae* and *Y. lipolytica* strains that metabolize phosphite using favorable raw materials (Shaw, Twilton & MacMillan, 2016). According to Silverman (2001), *Trichoderma* development or its biological properties are not affected by PTXD expression, resulting in a useful and observable phenotype for assessing the transcriptional function of the regulatory sequence. The metabolism of Phi gave *T. atroviride* a strategic advantage in overgrowth of harmful bacteria (Carreras-Villase nor et al., 2020). The phenotype remains vigorous and constant as transformants grow well even at high concentrations of Phi (Silverman, 2001). PTXD implements a simple enzymatic reaction involving the direct conversion of Phi to Pi using NAD + as a cofactor (Esland et al., 2018). The overall picture presented here can help in the development of fertilization strategies that maximize nutrient utilization, especially of P, while avoiding oversupply, which could reduce eutrophication of water bodies in case of wastewater discharge. This could be achieved by exploiting the physicochemical properties of Phi as Phi-metabolizing organisms (López-Arredondo & Herrera-Estrella, 2012). As a result, *ptxD*/Phi systems have the potential to minimize the cost of enzyme production using *Trichoderma*, as antibiotics and reactor sterilization are more expensive than Phi salts. The *ptxD*/Phi approach offers a more stable and cost-effective substitute for contamination control in the industrial development of *Trichoderma* and possibly other filamentous fungi, since Phi is FDA-approved for use as an agricultural fungicide and food additive (Carreras-Villase nor et al., 2020).

In addition, *ptxD*/Phi has been used for chloroplast transformation in *Chlamydomonas reinhardtii* and for the regulation of biological contaminants, expressing heterologous proteins in algal chloroplasts without affecting culture efficiency (Zhang, Wang & Wang, 2020). Consequently, *ptxD*/Phi selection can be used as a universal marker to transform *C. reinhardtii* wild-type microorganisms on medium containing Phi as a major source of P (Changko et al., 2020; Kanda et al., 2014). Combining phosphite fertilization with expression of the nuclear transgene *ptxD* provided an ideal substitute for herbicides in controlling weeds and pollution of algal cultures (Cutolo et al., 2020). Chloroplast expression of *ptxD* in *Chlamydomonas reinhardtii* has been proposed as a more environmentally friendly substitute for antibiotic resistance genes in plastid transformation (Day & Goldschmidt-Clermont, 2011). Transplastomic genotypes expressing the mutant PTXD type utilized NADP+ and NAD + to convert Phi to Pi more efficiently and evolved more rapidly than those expressing the wild-type protein (Cutolo et al., 2020). Loera-Quezada et al. (2016) reported that the production of genetically engineered microalgae capable of metabolizing Phi is a viable approach for developing a successful scheme for managing biological contaminants in microalgal development that can be combined with established biological and molecular strategies. The phosphite-based technique is important for closed photobioreactors in that one of the main advantages for Phi-metabolizing strains is that it eliminates the need for a sterile phase, which is the most expensive aspect of operating large photobioreactors (Loera-Quezada et al., 2016).

In addition, contamination by biological contaminants is a major obstacle to commercial microalgal processing using open culture systems (Lam et al., 2018). Due to low maintenance costs and relative ease of operation, Phi-based culture systems are ideal to meet the requirements of large-scale algal biomass production (Grobbelaar, 2012). Photosynthesis-dependent, phosphate-requiring CO_2_ metabolism could be more effectively maintained (Johnson & Alric, 2013), resulting in greater sugar and starch accumulation than the restricted wild-type enzyme, ultimately supporting more rapid selective development of algae in phi nutrient media. *C. reinhardtii* cells with mutant PTXD in their chloroplasts develop faster in phosphite media than cells expressing WT PTXD (Esland et al., 2018). As a result, Phi-based engineering will address contamination concerns in open pond systems and expand its application beyond microalgal species that can develop in harsh environments. Engineering Phi metabolism has biological consequences for current microalgal culture techniques, including: (i) the technique should allow closed photobioreactors to be operated at lower cost by removing the burden of sterilizing the media and reactor; (ii) the system should allow racecourse ponds to be used extensively for the development of microalgal biomass and derivatives; and (iii) the Phi scheme should allow the development of metabolic engineering in a variety of microalgal species.

## Conclusions

Plant transformation methods rely on the use of selection agents due to the low efficiencies of transformation. Traditionally, these selection agents usually contain antibiotic-resistant and herbicide-resistant genes, which have raised increasing public concern about the use of antibiotic-resistant and herbicide-selectable marker genes from a food safety and environmental perspective. This raises concern when genetically modified crops and their products become known to the public. In this scenario, concerted efforts have been made to search for alternative selection systems that are safe and reliable to produce transgenic crops to address public perception about the acceptability of GM crops and their products. In recent years, alternative selection markers, such as positively selectable markers containing either non-antibiotic resistance or native plant genes, have been evaluated for plant transformation. Such markers have been successfully used in a variety of plant species.

In addition, several other techniques such as co-transformation, site-specific recombination, intra-chromosomal homologous recombination, and transposon-based techniques have been used to develop transgenic plants to satisfy public concerns and to address other biosafety issues. However, the processes involved in these techniques are tedious, time consuming and not well understood. In the present scenario, *ptxD*/Phi could serve as a very successful scheme to suppress weeds under natural, low-phosphorus soils, even those resistant to the herbicide glyphosate, while allowing plants with *ptxD* expression to develop optimally due to reduced competition from incapacitated weeds. Production of genetically engineered microalgae and other microorganisms capable of metabolizing Phi is also a viable approach for managing biological contaminants. Therefore, *ptxD* transformants could be evaluated in soils with low phosphorus levels using Phi as herbicide. As a selective agent, *ptxD*/Phi is less toxic to untransformed cells compared to antibiotics, herbicides, or drugs and therefore appears to produce greater frequencies for transformation. The *ptxD*/Phi technology offers one of the most favorable techniques for transforming crops and other organisms with desired genes and ensures that various issues related to biosafety of GM products are minimized. Ultimately, it remains to be determined whether it can provide greater safety than the selectable marker genes already in use.

## Additional Information and Declarations

### Data Availability

The following information was supplied regarding data availability: There is no raw data or code in this review article.
